# Delayed Antibiotic Therapy and Organ Dysfunction in Critically Ill Septic Patients in the Emergency Department

**DOI:** 10.3390/jcm8020222

**Published:** 2019-02-08

**Authors:** Sung Yeon Hwang, Jikyoung Shin, Ik Joon Jo, Jong Eun Park, Hee Yoon, Won Chul Cha, Min Seob Sim, Tae Gun Shin

**Affiliations:** Department of Emergency Medicine, Samsung Medical Center, Sungkyunkwan University School of Medicine, Gangnam-gu, Seoul 06351, Korea; gerup@hanmail.net (S.Y.H.); shadow-jk@hanmail.net (J.S.); drjij@skku.edu (I.J.J.); jebbfirst@gmail.com (J.E.P.); wildhi.yoon@samsung.com (H.Y.); docchaster@gmail.com (W.C.C.); minsub01.sim@samsung.com (M.S.S.)

**Keywords:** sepsis, septic shock, multiple organ failure, antibacterial agents, timing

## Abstract

Background: We investigated the effect of antibiotic timing on outcomes based on changes in surrogate markers of organ failure, including platelet, serum bilirubin, serum creatinine levels, and the PaO_2_/FiO_2_ (P/F) ratio. Methods: This was a single-center, retrospective observational study of critically ill septic patients who presented to the emergency department (ED). The study period extended from August 2008 to September 2016. The primary outcomes included changes in platelet, serum bilirubin, serum creatinine levels, and the P/F ratio (δ-platelet, δ-serum bilirubin, δ-serum creatinine, and δ-P/F ratio were calculated as values measured on Day 3; values measured at ED enrollment). A multivariable linear regression model was developed to assess variables related to outcomes (δ-platelet, δ-serum bilirubin, δ-serum creatinine, and δ-P/F ratio). Results: We analyzed 1784 patients who met the inclusion criteria. The overall 28-day mortality was 14% (*n* = 256/1784). On multivariable linear regression analysis, the hourly delay in antibiotic therapy was significantly associated with a decrease in *δ*-platelet count (coefficient, −1.741; standard error, 0.740; p = 0.019), and an increase in *δ*-serum bilirubin (coefficient, 0.054; standard error, 0.021; p = 0.009). In contrast, it was not associated with *δ*-creatinine (coefficient, 0.008; standard error, 0.010; p = 0.434) or the δ-P/F ratio (coefficient, −0.797; standard error, 1.858; *p* = 0.668). Conclusion: The hourly delay of antibiotic therapy was associated with decreased platelet count and increased serum bilirubin concentration in critically ill septic patients during the first three days of ED admission.

## 1. Background

Sepsis and septic shock are life-threatening conditions that arise when a dysregulated response to a microbial infection injures a host’s own organs [1]. Sepsis affects millions of people worldwide, and is one of the leading causes of morbidity and mortality [2]. A recently updated definition of sepsis uses the Sequential Organ Failure Assessment (SOFA) score to clinically characterize organ dysfunction [3]. The SOFA score is made of six variables representing organ systems including the respiratory (PaO_2_/FiO_2_ (P/F) ratio), cardiovascular (mean arterial pressure and the use of inotropic agents or vasopressors), hepatic (bilirubin level), coagulation (platelet count), renal (creatinine level and urine output) and neurological (Glasgow coma scale (GCS)) systems.

Over the past two decades, early recognition and timely treatment have been the main considerations in the management of sepsis and septic shock. The early administration of antibiotics is one of the cornerstones of this management, and is widely used as a key index of quality of care. The Surviving Sepsis Campaign guidelines recommend broad-spectrum antibiotic therapy be initiated as soon as possible, but at least within one hour after the recognition of sepsis or septic shock [4,5].

The early administration of antibiotics is meant to reduce microbial burden, and therefore improve patient outcomes. The utility of early antibiotic therapy in patients with sepsis or septic shock is widely accepted. Numerous studies have investigated the association between timing of antibiotic therapy and outcomes. However, despite the strong hypothesis in favor of early antibiotic therapy, clinical evidence so far has been controversial. Therefore, there is ongoing uncertainty regarding the timing of antibiotic therapy.

In this study, we investigated the effect of antibiotic timing on the outcomes of sepsis based on changes in surrogate markers of organ failure, including the SOFA score components of platelet count, serum bilirubin and creatinine levels, and PaO_2_/FiO_2_ (P/F) ratio.

## 2. Materials and Methods

The study was approved by the institutional review board of Samsung Medical Center (IRB number, 2018-05-014-001). The need for informed consent was waived given the study’s retrospective, observational and anonymous nature. All methods were performed in accordance with relevant guidelines/regulations.

This was a single-center, retrospective study of critically ill septic patients who presented to the emergency department (ED) at Samsung Medical Center between August 2008 and September 2016. Samsung Medical Center is a tertiary referral hospital with an annual census of 70,000.

Patients who met all the following criteria were eligible for inclusion: (1) 18 years of age or older; (2) a diagnosis of sepsis with hyperlactatemia (≥4 mmol/L) or refractory hypotension during the ED stay. Sepsis was defined as an acute change in the total SOFA score ≥2 points, which was attributable to an infection. The baseline SOFA score was assumed to be zero in patients who were not known to have preexisting organ dysfunction. Refractory hypotension was defined by the following: persistent hypotension (systolic arterial pressure <90 mmHg, mean arterial pressure <60 mmHg); or a reduction in systolic blood pressure of more than 40 mmHg from baseline despite at least 1 L or more intravenous fluid challenge; or hypotension requiring vasopressors to maintain mean arterial pressure ≥65 mmHg.

The exclusion criteria were as follows: (1) patients who had previously signed a “Do Not Resuscitate” order; (2) patients with a terminal malignancy refractory to chemotherapy or radiation therapy; (3) patients who had set limitations on invasive care; (4) patients with missing data including platelet, bilirubin, and creatinine levels; (5) patients who received antibiotics ≥13 h after ED arrival or those in whom hyperlactatemia or refractory hypotension developed >6 h after ED arrival. These patients were excluded from the analysis in order to ensure homogeneity of the study population.

We used data of septic patients from an institutional registry. This registry has been used since 2008 to collect data and quality improvement activity in our ED. Until the new sepsis and septic shock definitions were released, we used the definition from the 1991 American College of Chest Physician and Society of Critical Care Medicine consensus conference [6]. After these new definitions were released, the SOFA criteria were applied to the previously collected data to facilitate communication in both clinical and research settings. 

The research coordinator periodically screened the electronic medical records (EMR) using the preset criteria. The coordinator also reviewed the preliminary lists of patients who were suspected of having sepsis or septic shock during their ED stay, as documented by the ED physicians. The demographic and clinical data of the eligible were abstracted from EMR by the coordinator: demographic characteristics including age, sex, and comorbidities; suspected infectious focus; blood cultures; laboratory data; therapeutic interventions, including antibiotic administration; initial fluid resuscitation; vasopressor use; renal replacement therapy; and 28-day mortality. The time to antibiotic therapy was calculated by subtracting the time of ED triage from the first time of broad-spectrum antibiotic administration (documented in the EMR). A resistant bacterial infection was defined as an isolated pathogen that exhibited resistance to initial antibiotics in antimicrobial susceptibility tests. SOFA scores were examined at the time of study enrollment, and again on Day 3. Acute Physiology and Chronic Health Evaluation (APACHE) II scores were calculated using the worst parameters measured within 24 h after ED arrival [7].

The primary outcomes included changes (δ) in the platelet count, serum bilirubin and creatinine levels, and P/F ratio on Day 3, which were calculated by subtracting the values measured at ED enrollment from the corresponding values obtained on Day 3. To determine the P/F ratio, the maximum values were collected. For cases with missing values, PaO_2_ was estimated using the peripheral oxygen saturation [8]. We used the nearest follow-up values among earlier measurements if patients died before Day 3, and classified patients as showing no change if they died prior to follow-up testing.

The continuous variables were presented as medians and interquartile ranges (IQR), and the Wilcoxon rank sum test was used for comparisons. The categorical data were presented as numbers with percentages, and compared using the chi-squared test. A multivariable linear regression model was developed to assess the variables related to outcomes (δ-platelet, δ-serum bilirubin, δ-serum creatinine, and δ-P/F ratio). Potential confounding variables were selected a priori by univariable regression analysis with a threshold of *p* < 0.20. With regard to the δ-platelet, those who had received platelet transfusions were excluded from the analysis. Similarly, patients who received renal replacement therapy were excluded from the δ-creatinine analysis. The effect of multicollinearity was assessed using the variation inflation factor. *P*-values < 0.05 were considered statistically significant. Statistical analysis was performed using SAS version 9.4 (SAS Institute, Cary, NC, USA) and STATA version 15.1 (STATA Corporation, College Station, TX, USA).

## 3. Results

### 3.1. Baseline Characteristics

**Figure 1:**
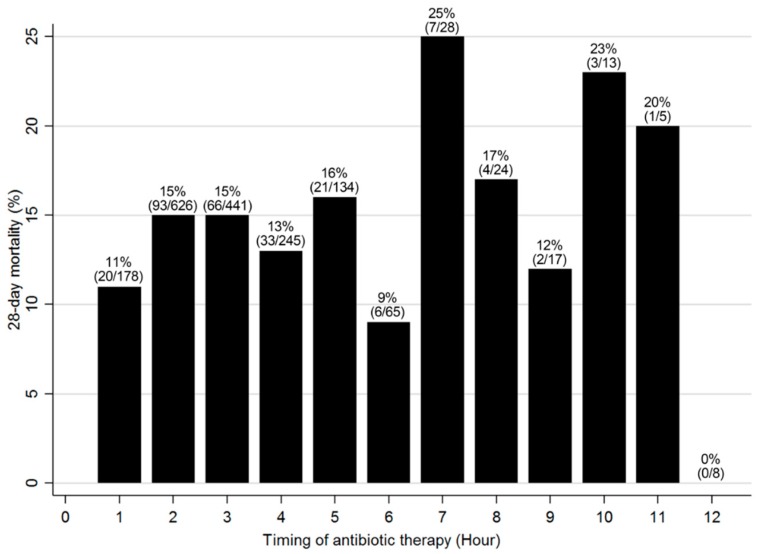
Crude 28-day mortality according to the timing of first antibiotic therapy. A total of 1784 patients were analyzed the crude mortality was 14%. The bars represent the proportions of patients who received their first antibiotic therapy within the indicated time period and died within 28 days after presentation to the emergency department. The number represents % (no./total no. of each group).

A total of 2636 septic patients were screened for this study. Of these, 852 patients were excluded for the following reasons: terminal malignancies (*n* = 200); “Do Not Attempt Resuscitation” order or limitations on invasive care (*n* = 390); missing data (*n* = 5); and antibiotics administration ≥13 h from ED triage, or diagnosis of sepsis >6 h after ED arrival (*n* = 257). Finally, we analyzed 1784 patients who met the inclusion criteria. The overall 28-day mortality was 14% (*n* = 256/1784). The 28-day mortality, according to the timing of antibiotic therapy, is shown in Figure 1. 

The baseline characteristics of each group are described in Table 1. Age and sex were not significantly different between the two groups. Chronic liver disease and metastatic solid cancers were more frequent in the non-survivor group than they were in the survivor group. The initial SOFA score was significantly higher in the non-survivor group (6, IQR 4–8 vs. 9, IQR 6–11, *p* < 0.001) than it was in the survivor group. In addition, fluid resuscitation within three hours, the use of vasopressors, and renal replacement therapy were all more frequent in the non-survivor group than they were in the survivor group.

**Figure 2:**
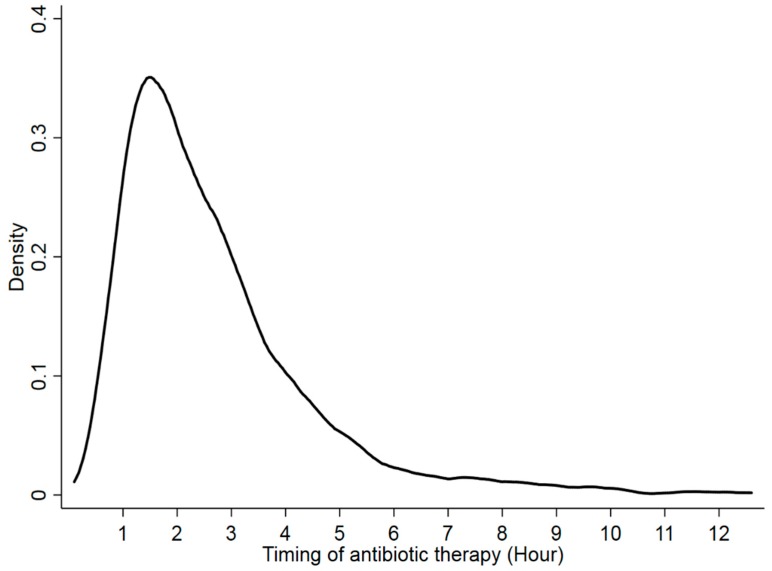
Kernel density plot of the time to first antibiotic therapy after triage in the emergency department. Overall, the median time to first antibiotic therapy was 2.2 h (interquartile range, 1.5, 3.3).

The median time to antibiotic therapy was 2.2 h (IQR, 1.4–3.3) in the survivor group and 2.3 h (IQR, 1.5–3.4 h) in the non-survivor group (*p* = 0.473) (Figure 2).

### 3.2. Outcome Comparison

Platelet count was significantly lower in the non-survivor group than it was in the survivor group (*p* < 0.001) on ED enrollment (Table 2). Although both groups exhibited a decreased platelet count on Day 3, this value remained significantly lower in the non-survivor group (*p* < 0.001). Serum bilirubin was significantly higher in the non-survivor group than it was in the survivor group on ED enrollment (*p* = 0.013). Serum bilirubin levels decreased in the survivor group, while they increased in the non-survivor group on Day 3. Serum creatinine was higher in the non-survivor group than it was in the survivor group on ED enrollment (*p* < 0.001). Serum creatinine decreased on Day 3 in both groups, but it was still higher in the non-survivor group than in the survivor group (*p* < 0.001). The P/F ratio remained higher in the survivor group than in the non-survivor group at both ED enrollment and Day 3 (*p* < 0.001 and *p* < 0.001, respectively).

**Figure 3:**
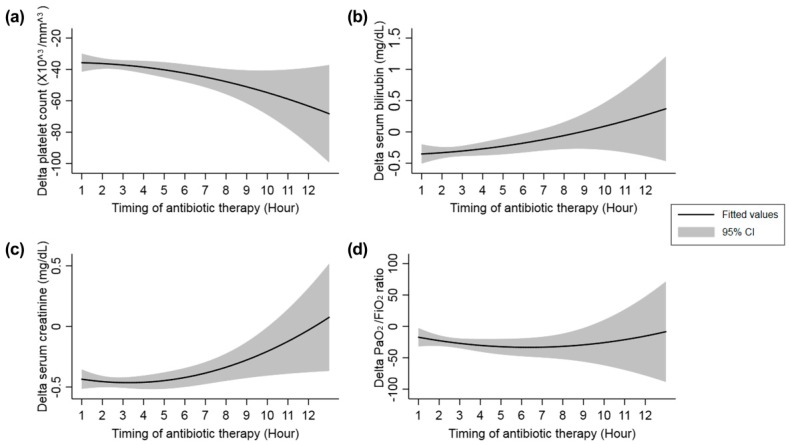
Changes in the measured parameters according to the timing of first antibiotic therapy. (**a**) δ-Platelet count, (**b**) δ-serum bilirubin level, (**c**) δ-serum creatinine level, and (**d**) δ- PaO_2_/FiO_2_ ratio. The δ-platelet count tended to decrease with increasing delays in the first antibiotic treatment after triage in the emergency department. In other words, a longer delay in the first antibiotic therapy led to a greater decrease in the platelet count on day 3. On the other hand, the δ-serum bilirubin and δ-serum creatinine levels on day 3 tended to increase with increasing delays in the first antibiotic therapy. The δ-PaO_2_/FiO_2_ ratio relatively remained constant. The total number of patients were 1525 for δ-platelet analysis, 1784 for δ-bilirubin, 1604 for δ-creatinine and 1780 for δ-PaO_2_/FiO_2_ ratio. CI, confidence interval.

Fitted lines with 95% confidence intervals showing the changes in δ-platelet count, δ-serum bilirubin, δ-serum creatinine, and δ-PaO_2_/FiO_2_ ratio according to the timing of first antibiotic therapy are shown in Figure 3.

### 3.3. Multivariable Linear Regression Analysis

Univariable analysis for each outcome is elsewhere (see Supplementary Tables S1–S4). Multivariable linear regression analysis is shown in Table 3. An hourly delay in antibiotic therapy was found to associate significantly with a lower δ-platelet count (coefficient, −1.741; standard error, 0.740; *p* = 0.019) and higher δ-serum bilirubin level (coefficient, 0.054; standard error, 0.021; *p* = 0.009). These findings suggest that delayed antibiotic administration correlates respectively with a decrease in the platelet count and an increase in serum bilirubin. However, no significant association were observed between an hourly delay in antibiotic therapy and either the δ-serum creatinine (coefficient, 0.005; standard error, 0.010; *p* = 0.434) or δ-P/F ratio (coefficient, −0.797; standard error, 1.858; *p* = 0.668). 

In addition to the time to antibiotic therapy, various clinical variables were found to associate with changes in the indicated markers. Interestingly, of the initial management variables, fluid resuscitation within 3 h (30 mL/kg) was associated with a lower δ-serum creatinine level (coefficient, −0.176; standard error, 0.042; *p* < 0.001) and higher δ-P/F ratio (coefficient, 25.775; standard error, 7.675; *p* = 0.001). However, the total fluid input within 24 h after ED arrival was associated with a lower δ-platelet count (coefficient, −4.225; standard error, 0.740; *p* < 0.001) and δ-P/F ratio (coefficient, −8.807; standard error, 1.862; *p* < 0.001) and an increase in the δ-serum bilirubin level (coefficient, 0.042; standard error, 0.021; *p* = 0.033). Similar to the total fluid input, the initial lactate level was also found to correlate positively with organ failure. The use of vasopressors on Day 1 was associated with an increase in the serum bilirubin level (coefficient, 0.147; standard error, 0.100; *p* = 0.031) and a lower δ-P/F ratio (coefficient, −21.697; standard error, 7.916; *p* = 0.006). 

On the other hand, some variables revealed associations that differed clinically depending on the surrogate markers. For instance, the initial C-reactive protein (CRP) level was found to associate with the δ-platelet count (coefficient, 0.571; standard error, 0.125; *p* < 0.001), δ-serum creatinine level (coefficient, −0.014; standard error, 0.002; *p* < 0.001), and δ-P/F ratio (coefficient, −1.217; standard error, 0.307; *p* < 0.001), but not with the δ-serum bilirubin level.

## 4. Discussion

In this study, we investigated the impact of antibiotic timing on outcomes assessed based on changes in surrogate markers of organ failure. We found that when antibiotic administration was delayed after ED triage, platelet count was decreased, and serum bilirubin was increased. The degree of serum creatinine and the P/F ratio change was not affected by antibiotic timing.

Numerous studies have evaluated the association between antibiotic timing and outcomes in septic patients. Regardless, there remains debate surrounding this issue [9,10,11,12,13,14,15]. Previously, we demonstrated that antibiotic therapy within three hours of ED arrival was significantly associated with improved outcomes in critically ill septic patients, including in-hospital survival, reversal of organ failure, and decreased hospital length of stay [15]. In this study, we focused on surrogate markers of organ failure that have been incorporated into the illness severity scoring system [16]. These findings provide additional supportive evidence regarding early antibiotic therapy from the point of view of organ failure.

Thrombocytopenia, which is usually defined as a platelet count of <150 × 10^3^/mm^3^, is prevalent among critically ill patients, and is a well-known biomarker of disease severity and mortality [17,18]. Akca et al. [17] conducted a prospective, multicenter, observational cohort study to investigate the time course of changes in the daily platelet count in critically ill ICU patients. The group found that the platelet count was acutely decreased in the early part of patient’s hospital courses. At any point measured, thrombocytopenia was associated with an increase in mortality. The risk of death was also higher if thrombocytopenia persisted for a prolonged period. Similarly, we found that the platelet counts were decreased on Day 3 after ED presentation, regardless of survival. Our findings suggest that delayed antibiotic therapy is associated with lower platelet count, and that early antibiotic therapy would delay progression of thrombocytopenia.

In this study, we found that the serum bilirubin level increases with each hour of delayed antibiotic therapy. Serum bilirubin is widely used as a surrogate marker of hepatic dysfunction in critically-ill patients [16]. In septic patients, the serum bilirubin may be elevated not only from hepatic dysfunction, but also from hemolysis and cholestasis [19]. Previous studies found that elevated serum bilirubin was independently associated with increased risk of sepsis related to ARDS and mortality in critically ill patients [20,21,22]. Even mild elevations in the serum bilirubin concentration (>1 mg/dL) were associated with increased mortality in critically ill patients without an obvious cause of hyperbilirubinemia [21]. Elevated bilirubin not only reflects the severity of the disease, but also plays a crucial role in the process of disease progression. For instance, Arai et al. [23] found that bilirubin impairs the bactericidal activity of neutrophils through an antioxidant mechanism.

Serum creatinine is used as a marker to classify acute kidney injury in various definition systems [24,25,26]. Both elevations and declines in serum creatinine are associated with poor prognosis in critically ill patients [27,28]. Serum creatinine concentration after ED presentation was not affected by antibiotic timing in our study. Serum creatinine can be difficult to interpret, as it can be acutely influenced by a number of factors [29]. There may be other confounding factors that have more influence on serum creatinine than antibiotic timing. In addition, our evaluation of the serum creatinine may have been too soon to detect changes related to delayed antibiotic administration. Therefore, it remains to be determined whether the serum creatinine level is affected by antibiotic timing.

The time to appropriate antimicrobial therapy has been identified as an independent determinant of a patient’s outcomes [30,31]. In an additional analysis, we found that initial antimicrobial resistance might be associated with a longer hospital length of stay, consistent with the findings of a previous study [30]. However, our lack of ability to identify a significant association between the isolation of a pathogen resistant to initial antibiotic therapy and the main outcomes might be attributable to several factors. First, this study did not consider patients without isolated pathogens, and the number of patients with resistant pathogens was too small to enable a full evaluation of the impact of this factor on the outcomes. Accordingly, this is a limitation of our study. Second, in many cases, physicians may either rapidly escalate the level of antibiotic therapy or administer additional antibiotics without a significant time delay. Therefore, patients who received initial antibiotics at an early time point might quickly receive a revised prescription. Third, the initial antibiotics might have some effect, despite the results of in vitro susceptibility tests.

Earlier antibiotic use might reduce the pathogen burden and modify the host response. This may explain the previous observation of less severe subsequent organ dysfunction in patients who rapidly received antibiotics [32]. Moreover, the core components of initial sepsis management were likely delivered rapidly to these patients after the prompt recognition of sepsis. In our study, we evaluated fluid resuscitation in addition to the sepsis bundle. Interestingly, early fluid resuscitation within 3 h was associated with favorable changes of in the serum creatinine level and P/F ratio. By contrast, the total fluid input within 24 h after ED arrival was associated with harmful effects on the platelet count, serum bilirubin level, and P/F ratio. These findings should be interpreted cautiously because the physiologic effects of fluid administration vary among individual patients and may be susceptible to confounders. Overall, however, these findings appear to support both the importance of early fluid resuscitation and the risk of fluid overload.

In multivariable analyses, we observed that some variables, including CRP, formed different associations to different surrogate markers. Although the underlying biologic mechanism remains uncertain, we think that a higher level of inflammatory severity might not always indicate worse outcomes and that the trends of changes in organ failure over the indicated time interval might differ from those of single values. In addition, each marker of organ failure might correlate more strongly with other factors such as comorbidities and infection focus.

Organ failure can be assessed using various laboratory tests and clinical findings. In this study, we used four readily available variables but did not evaluate other surrogate indexes, including the GCS and the reversal of shock. We note that it might be difficult to evaluate the GCS in septic shock patients under sedation or mechanical ventilation at high levels of accuracy and interrater reliability. Therefore, we think that a direct comparison of the GCS might have yielded inappropriate results in this retrospective study. Furthermore, approximately 40% of patients in our study cohort did not receive vasopressors during the first day. Therefore, further studies of circulatory failure in a larger population of patients with septic shock might require different methods and a focus on shock reversal.

This study has several limitations. First, it was a retrospective study with associated inherent biases based on the study design. We were unable to establish any causal relationships based on our observations. We were also unable to identify why antibiotic administration was delayed (i.e. delayed recognition of sepsis, delayed preparation of antibiotics, or delayed delivery). Another limitation is that our results may not be generalizable to the overall population given that this study was conducted at a single center. Third, we did not evaluate the entire clinical course of shock in terms of the primary resolution of the infection source and the appearance of new pathogens or infection sources during the course of a hospital stay. Fourth, we did not evaluate patients who received antibiotics ≥13 h after ED. These patients comprised ≤2% of our initial study cohort, and may have been outliers likely to experience diagnostic uncertainty or to have received delayed treatment [11,32]. Finally, we could not collect accurate data for patients with early death and used some estimated values. We thought that excluding these patients was not appropriate based on the clinical context, although this may have led to some underestimation or overestimation of change in values. When we performed additional analysis of data excluding these patients, the results were similar.

## 5. Conclusions

Hourly delay of antibiotic therapy was associated with decreased platelet count and increased serum bilirubin concentration during the initial three days after ED arrival in critically ill septic patients. In contrast, the degree of serum creatinine and the P/F ratio change was not affected by the timing of antibiotic therapy. These findings suggested there is at least some benefit of early antibiotic therapy in septic patients with regard to organ failure.

## Figures and Tables

**Table 1:** Baseline characteristics.

Variables	Overall (*n* = 1784)	Survivor Group (*n* = 1528)	Non-Survivor Group (*n* = 256)	*p*-Value
Age (years)	66 (55, 73)	66 (55, 73)	66 (55, 73)	0.656
Sex (male)	1027 (57.6)	873 (57.1)	154 (60.2)	0.365
Comorbidities				
Hypertension	633 (35.5)	549 (35.9)	84 (32.8)	0.335
Diabetes	450 (25.2)	391 (25.6)	59 (23.1)	0.386
Cardiac disease	227 (12.7)	189 (12.4)	38 (14.8)	0.272
Chronic lung disease	109 (6.1)	90 (5.9)	19 (7.4)	0.344
Chronic renal disease	92 (5.2)	80 (5.2)	12 (4.7)	0.714
Chronic liver disease ^†^	157 (8.8)	122 (8.0)	35 (13.7)	0.003
Metastatic solid cancer ^†^	406 (22.8)	318 (20.8)	88 (34.4)	<0.001
Hematologic malignancy	185 (10.4)	155 (10.1)	30 (11.7)	0.444
Suspected infectious focus ^†^				<0.001
Intra-abdominal	595 (33.4)	526 (34.4)	69(27.0)	
Respiratory	635 (35.6)	508 (33.3)	127 (49.6)	
Urinary	234 (13.1)	226 (14.8)	8 (3.1)	
Other	320 (17.9)	268 (17.5)	52 (20.3)	
Positive blood cultures	728 (40.8)	621 (40.6)	107 (41.8)	0.728
Resistant bacterial infection	147 (8.2)	131 (8.6)	16 (6.3)	0.211
SOFA score (Baseline) ^†^	6 (4, 9)	6 (4, 8)	9 (6, 11)	<0.001
SOFA score (Day 3) ^†^	5 (3, 8)	4 (3, 7)	10 (7, 14)	<0.001
APACHE II score (Baseline) ^†^	18 (13, 24)	17 (12, 23)	21 (15, 30)	<0.001
Laboratory results				
Initial lactate (mmol/L) ^†^	4.3 (2.7, 5.6)	4.2 (2.6, 5.4)	5.3 (3.5, 8.4)	<0.001
C-reactive protein (mg/dL) ^†^	10.7 (4.4, 21.6)	9.9 (3.9, 20.5)	15.2 (8.2, 25.9)	<0.001
Interventions				
Fluid resuscitation within 3 h (30 mL/kg) ^†^	873 (49.0)	727 (47.6)	146 (57.0)	0.005
Fluid input within 24 h (L) ^†^	4.3 (3.0, 5.7)	4.2 (3.0, 5.5)	5.0 (3.5, 6.5)	<0.001
Vasopressors ^†^	1111 (62.3)	902 (59.0)	209 (81.6)	<0.001
Renal replacement therapy ^†^	179 (10.0)	104 (6.8)	75 (29.3)	<0.001
Time to antibiotic therapy (hours) *	2.2 (1.5, 3.3)	2.2 (1.4, 3.3)	2.3 (1.5, 3.4)	0.473

A total of 1784 patients were analyzed. Continuous variables are presented as medians (25th percentile, 75th percentile) and were compared using the Wilcoxon rank sum test. Categorical data are presented as numbers (%) and were compared using the chi-squared test. * The time to antibiotic therapy was calculated by subtracting the emergency department triage time from the first time of broad-spectrum antibiotics administration. ^†^ *p*-value <0.05 was considered statistically significant. SOFA, Sequential Organ Failure Assessment; APACHE, Acute Physiology and Chronic Health Evaluation.

**Table 2:** Comparing serum platelet, bilirubin, and creatinine levels across groups.

	Overall (*n* = 1784)	Survivor Group (*n* = 1528)	Non-Survivor Group (*n* = 256)	*p*-Value
Platelet (× 10^3^/mm^3^) *				
ED enrollment ^†^	143 (73, 216)	150 (80, 217)	102 (40, 178)	<0.001
Day 3 ^†^	100 (49, 171)	108 (56, 177)	52 (27.5, 110.5)	<0.001
δ-platelet count	−27 (−63, −1)	−27 (−61, −2)	−28.5 (−77, 0)	0.262
Serum bilirubin (mg/dL) *				
ED enrollment ^†^	1.2 (0.8, 2.2)	1.2 (0.8, 2.1)	1.4 (0.8, 2.7)	0.013
Day 3 ^†^	0.9 (0.6, 1.7)	0.9 (0.5, 1.5)	1.55 (0.8, 3.7)	<0.001
δ-serum bilirubin ^†^	−0.3 (−0.7, 0)	−0.3 (−0.8, 0)	0 (−0.3, 0.8)	<0.001
Serum creatinine (mg/dL) *				
ED enrollment ^†^	1.26 (0.91, 1.87)	1.22 (0.88, 1.8)	1.48 (1.05, 2.38)	<0.001
Day 3 ^†^	0.85 (0.62, 1.31)	0.81 (0.6, 1.16)	1.39 (0.9, 1.92)	<0.001
δ-serum creatinine ^†^	−0.31 (−0.61, −0.12)	−0.33 (−0.62, −0.15)	−0.13 (−0.47, 0.05)	<0.001
PaO_2_/FiO_2_ ratio *				
ED enrollment ^†^	342 (250, 429)	348 (260, 436)	289 (166, 409)	<0.001
Day 3 ^†^	321 (207, 410)	343 (232, 410)	166 (90, 291)	<0.001
δ-PaO_2_/FiO_2_ ratio ^†^	−11 (−103, 55)	−2 (−89, 60)	−68 (−189, 19)	<0.001

Data are presented as medians (25th percentile, 75th percentile) and were compared using the Wilcoxon rank sum test. * The total number of patients were 1525 for δ-platelet analysis after excluding if they had received platelet transfusions, 1784 for δ-bilirubin analysis, 1604 for δ-creatinine analysis after excluding if they received renal replacement therapy and 1780 for δ-PaO_2_/FiO_2_ ratio analysis after excluding patients with missing values. ^†^ *p*-value < 0.05 was considered statistically significant. ED, emergency department; PaO_2_, partial pressure of oxygen; FiO_2_, fraction of inspired oxygen.

**Table 3:** Multivariable linear regression analysis.

Variables	Regression Coefficient	Standard Error	*p*-Value
Analysis of δ-Platelet Count (×10^3^/mm^3^) *			
Timing of antibiotic therapy (per hour delay ^†^)	−1.950	0.749	0.009
Age	−0.140	0.101	0.164
Chronic renal disease ^†^	−18.546	6.214	0.003
Chronic liver disease ^†^	26.021	5.086	<0.001
Hematologic malignancy ^†^	16.257	4.899	0.001
Initial lactate (mmol/L) ^†^	−2.764	0.473	<0.001
C-reactive protein (mg/dL) ^†^	0.571	0.125	<0.001
APACHE II score ^†^	−0.381	0.171	0.026
Use of vasopressors within 24 h	−0.552	3.156	0.861
Fluid input within 24 h (L) ^†^	−4.225	0.740	<0.001
Suspected infectious focus			
Intra-abdominal	Reference		
Respiratory ^†^	13.866	3.382	<0.001
Urinary ^†^	15.782	4.495	<0.001
Other ^†^	16.206	4.093	<0.001
Analysis of δ-Serum Bilirubin (mg/dL) *	**Regression Coefficient**	**Standard Error**	***p*-Value**
Timing of antibiotic therapy (per hour delay) ^†^	0.056	0.021	0.009
Hematologic malignancy ^†^	0.450	0.126	<0.001
Metastatic solid cancer ^†^	0.167	0.090	0.046
Initial lactate (mmol/L) ^†^	0.081	0.013	<0.001
C-reactive protein (mg/dL)	0.001	0.003	0.409
Blood culture-positive	−0.129	0.081	0.117
Resistant bacterial infection	−0.170	0.137	0.341
APACHE II score	0.015	0.015	0.563
Use of vasopressors within 24 h ^†^	0.147	0.100	0.031
Fluid resuscitation within 3 h (30 mL/kg)	−0.058	0.084	0.924
Fluid input within 24 h (L) ^†^	0.042	0.021	0.033
Suspected infectious focus			
Intra-abdominal	Reference		
Respiratory ^†^	0.294	0.093	0.001
Urinary ^†^	0.298	0.121	0.007
Other ^†^	0.244	0.109	0.012
Analysis of δ-Serum Creatinine (mg/dL) *	**Regression Coefficient**	**Standard Error**	***p*-Value**
Timing of antibiotic therapy (per hour delay)	0.005	0.010	0.434
Cardiac disease	0.079	0.058	0.258
Chronic lung disease	0.167	0.080	0.371
Hematologic malignancy ^†^	0.150	0.064	0.017
Initial lactate (mmol/L)	−0.020	0.008	0.092
C-reactive protein (mg/dL) ^†^	−0.014	0.002	<0.001
APACHE II score ^†^	0.001	0.008	<0.001
Use of vasopressors within 24 h	−0.063	0.050	0.095
Fluid resuscitation within 3 h (30 mL/kg) ^†^	−0.176	0.042	<0.001
Fluid input within 24 h (L)	−0.015	0.011	0.725
Suspected infectious focus	0.005		
Intra-abdominal	0.079		
Respiratory ^†^	0.167	0.047	<0.001
Urinary	0.150	0.061	0.413
Other	−0.020	0.056	0.403
Analysis of δ-PaO_2_/FiO_2_ Ratio *	**Regression Coefficient**	**Standard Error**	***p*-Value**
Timing of antibiotic therapy (per hour delay)	−0.797	1.858	0.668
Hypertension	3.328	7.426	0.654
Diabetes	11.740	8.201	0.152
Cardiac disease	7.785	10.228	0.447
Metastatic solid cancer ^†^	−18.769	8.092	0.020
Initial lactate (mmol/L) ^†^	−5.900	1.187	<0.001
C-reactive protein (mg/dL) ^†^	−1.217	0.307	<0.001
Blood culture-positive ^†^	−16.016	7.242	0.027
Resistant bacterial infection	7.760	12.365	0.530
APACHE II score ^†^	2.509	0.406	<0.001
Use of vasopressors within 24 h ^†^	−21.697	7.961	0.006
Fluid resuscitation within 3 h (30 mL/kg) ^†^	25.775	7.675	0.001
Fluid input within 24 h (L) ^†^	−8.807	1.862	<0.001
Suspected infectious focus			
Intra-abdominal	Reference		
Respiratory	8.038	8.449	0.342
Urinary	20.069	10.931	0.067
Other	7.022	9.821	0.475

Multivariable linear regression analysis was used to assess the variables related to outcomes (δ-platelet, δ-serum bilirubin, δ-serum creatinine, and δ-P/F ratio). Potential confounding variables were selected a priori using a univariable regression analysis with a threshold of *p* < 0.20. The mean variance inflation factors were 1.17 for δ-platelet count, 1.20 for δ-serum bilirubin, 1.14 for δ-serum creatinine, and 1.19 for δ-PaO_2_/FiO_2_ ratio. *The total number of patients were 1525 for δ-platelet analysis after excluding if they had received platelet transfusions, 1784 for δ-bilirubin analysis, 1604 for δ-creatinine analysis after excluding if they received renal replacement therapy and 1780 for δ-PaO_2_/FiO_2_ ratio analysis after excluding patients with missing values. ^†^ *p*-value < 0.05 was considered statistically significant. APACHE, Acute Physiology and Chronic Health Evaluation; PaO_2_, partial pressure of oxygen; FiO_2_, fraction of inspired oxygen.

## Supplementary Materials

The following are available online at http://www.mdpi.com/2077-0383/8/2/222/s1, Table S1: Univariable regression analysis of the δ-platelet count; Table S2: Univariable regression analysis of the δ-serum bilirubin. Table S3: Univariable regression analysis of the δ-serum creatinine. Table S4: Univariable regression analysis of the δ- PaO2/FiO2 ratio.
