# Supplementary material for: Delayed Antibiotic Therapy and Organ Dysfunction in Critically Ill Septic Patients in the Emergency Department
Source: J Clin Med. 2019 Feb 8;8(2):222. doi: 10.3390/jcm8020222 (PMC6406307; doi:10.3390/jcm8020222)
Supplement: Supplementary file 1 [file jcm-08-00222-s001.pdf]

Supplementary Table S1. Univariable regression analysis of the δ-platelet count

**Variables**	**Regression coefficient**	**Standard error**	***p***
Timing of antibiotic therapy (per hour delay)†	-1.860	0.771	0.016
Age (years)†	-0.212	0.101	0.035
Sex (male)	2.494	2.861	0.385
Comorbidities			
Hypertension	-1.975	2.952	0.504
Diabetes	4.095	3.253	0.209
Cardiac disease	-4.719	4.233	0.265
Chronic lung disease	-2.670	5.879	0.650
Chronic renal disease†	-16.900	6.364	0.008
Chronic liver disease†	14.784	5.131	0.004
Metastatic solid cancer	-1.237	3.371	0.714
Hematologic malignancy†	18.407	4.969	<0.001
Suspected infectious focus			
Intra-abdominal	Reference		
Respiratory†	15.710	3.376	<0.001
Urinary†	14.477	4.603	0.002
Other†	14.377	4.152	0.001
Positive blood cultures	0.253	2.885	0.930
Resistant bacterial infection	-5.121	5.142	0.319
APACHE II score†	-0.391	0.163	0.016
Laboratory results			
Initial lactate (mmol/L)†	-3.199	0.480	<0.001
C-reactive protein (mg/dL)†	0.386	0.125	0.002
Interventions			
Fluid resuscitation within 3 hours (30 ml/kg)	-2.399	2.835	0.398
Fluid input within 24 hours (L)†	-4.166	0.671	<0.001
Vasopressors†	-6.869	2.913	0.019

The total number of patients was 1525 for δ-platelet analysis after excluding if they had received platelet transfusions. Univariable linear regression analysis was used. †P-value <0.05 was considered statistically significant. APACHE, Acute Physiology and Chronic Health Evaluation.

Supplementary Table S2. Univariable regression analysis of the δ-serum bilirubin

**Variables**	**Regression coefficient**	**Standard error**	***P***
Timing of antibiotic therapy (per hour delay)†	0.044	0.021	0.031
Age (years)	-0.003	0.003	0.282
Sex (male)	0.088	0.077	0.249
Comorbidities			
Hypertension	0.025	0.079	0.755
Diabetes	-0.041	0.087	0.640
Cardiac disease	0.058	0.114	0.611
Chronic lung disease	-0.149	0.158	0.344
Chronic renal disease	0.196	0.171	0.251
Chronic liver disease	0.121	0.134	0.367
Metastatic solid cancer	0.157	0.090	0.082
Hematologic malignancy†	0.510	0.124	<0.001
Suspected infectious focus			
Intra-abdominal†	Reference		
Respiratory	0.349	0.091	<0.001
Urinary	0.237	0.123	0.055
Other†	0.370	0.110	0.001
Positive blood cultures	-0.105	0.077	0.173
Resistant bacterial infection†	-0.281	0.137	0.041
APACHE II score†	0.025	0.004	<0.001
Laboratory results			
Initial lactate (mmol/L)†	0.084	0.013	<0.001
C-reactive protein (mg/dL)	0.006	0.003	0.088
Interventions			
Fluid resuscitation within 3 hours (30 ml/kg)	0.099	0.076	0.192
Fluid input within 24 hours (L)†	0.068	0.018	<0.001
Vasopressors†	0.261	0.078	0.001

The total number of patients was 1784. Univariable linear regression analysis was used. †P-value <0.05 was considered statistically significant. APACHE, Acute Physiology and Chronic Health Evaluation.

Supplementary Table S3. Univariable regression analysis of the δ-serum creatinine

**Variables**	**Regression coefficient**	**Standard error**	***p***
Timing of antibiotic therapy (per hour delay)	0.018	0.011	0.100
Age (years)	0.001	0.001	0.491
Sex (male)	0.030	0.040	0.453
Comorbidities			
Hypertension	-0.010	0.042	0.804
Diabetes	0.036	0.046	0.429
Cardiac disease	0.100	0.060	0.098
Chronic lung disease†	0.202	0.081	0.013
Chronic renal disease	0.090	0.121	0.460
Chronic liver disease	-0.013	0.071	0.856
Metastatic solid cancer	-0.014	0.046	0.762
Hematologic malignancy	0.112	0.065	0.085
Suspected infectious focus			
Intra-abdominal	Reference		
Respiratory†	0.185	0.047	<0.001
Urinary	-0.074	0.063	0.244
Other	0.063	0.057	0.272
Positive blood cultures	-0.028	0.040	0.484
Resistant bacterial infection	-0.031	0.072	0.667
APACHE II score†	-0.005	0.002	0.029
Laboratory results			
Initial lactate (mmol/L)	-0.015	0.008	0.052
C-reactive protein (mg/dL)†	-0.015	0.002	<0.001
Interventions			
Fluid resuscitation within 3 hours (30 ml/kg †	-0.230	0.039	<0.001
Fluid input within 24 hours (L)†	-0.051	0.009	<0.001
Vasopressors†	-0.185	0.040	<0.001

The total number of patients was 1604 after excluding if they received renal replacement therapy. Univariable linear regression analysis was used. †P-value <0.05 was considered statistically significant. APACHE, Acute Physiology and Chronic Health Evaluation.

Supplementary Table S4. Univariable regression analysis of the δ- PaO_2/FiO_2 ratio

**Variables**	**Regression coefficient**	**Standard error**	***p***
Timing of antibiotic therapy (per hour delay)			
Age (years)	0.217	0.244	0.374
Sex (male)	-7.811	6.963	0.262
Comorbidities			
Hypertension	12.497	7.181	0.082
Diabetes†	19.039	7.917	0.016
Cardiac disease†	22.246	10.321	0.031
Chronic lung disease	15.581	14.412	0.280
Chronic renal disease	8.324	15.487	0.591
Chronic liver disease	-3.122	12.211	0.798
Metastatic solid cancer†	-19.151	8.231	0.020
Hematologic malignancy	-0.242	11.267	0.983
Suspected infectious focus			
Intra-abdominal	Reference		
Respiratory†	19.156	8.288	0.021
Urinary	29.727	11.161	0.008†
Other	13.946	10.060	0.166
Positive blood cultures†	-27.617	6.979	<0.001
Resistant bacterial infection	15.794	12.589	0.210
APACHE II score†	1.884	.387	<0.001
Laboratory results			
Initial lactate (mmol/L)†	-6.100	1.186	<0.001
C-reactive protein (mg/dL)	-1.301	0.304	<0.001
Interventions			
Fluid resuscitation within 3 hours (30 ml/kg)†	16.396	6.884	0.017
Fluid input within 24 hours (L)†	-9.503	1.636	<0.001
Vasopressors†	-24.218	7.079	0.001

The total number of patients was 1780 after excluding patients with missing values. Univariable linear regression analysis was used. †P-value <0.05 was considered statistically significant. APACHE, Acute Physiology and Chronic Health Evaluation.
